# Specific visual expertise reduces susceptibility to visual illusions

**DOI:** 10.1038/s41598-025-88178-y

**Published:** 2025-03-13

**Authors:** Radoslaw Wincza, Calum Hartley, Tim Donovan, Sally Linkenauger, Trevor Crawford, Debra Griffiths, Martin Doherty

**Affiliations:** 1 Lancaster University, Lancaster, UK; 2 University of Central Lancashire, Preston, UK; 3 Cumbria University, Lancaster, UK; 4 University of East Anglia, Norwich, UK

**Keywords:** Medical image perception, Context integration, Neuroplasticity, Size constancy mechanisms, Visual illusions, Human behaviour, Medical research

## Abstract

Extensive exposure to specific kinds of imagery tunes visual perception, enhancing recognition and interpretation abilities relevant to those stimuli (e.g. radiologists can rapidly extract important information from medical scans). For the first time, we tested whether specific visual expertise induced by professional training also affords domain-general perceptual advantages. Experts in medical image interpretation (*n* = 44; reporting radiographers, trainee radiologists, and certified radiologists) and a control group consisting of psychology and medical students (*n* = 107) responded to the Ebbinghaus, Ponzo, Müller-Lyer, and Shepard Tabletops visual illusions in forced-choice tasks. Our results show that medical image experts were significantly less susceptible to all illusions except for the Shepard Tabletops, demonstrating superior perceptual accuracy. These findings could possibly be attributed to a stronger local processing bias, a by-product of learning to focus on specific areas of interest by disregarding irrelevant context in their domain of expertise.

**Supplementary Information:**

The online version contains supplementary material available at 10.1038/s41598-025-88178-y.

## Introduction

Expertise is the culmination of a lengthy and deliberate process of acquiring and mastering a specific skill^1^. Domains necessitating extensive visual expertise include face processing^2^, chess^3^, and radiology^4,5^. This study focuses on expertise in medical image interpretation, specifically radiology and radiography. Much attention has been allocated to the study of global perception in visual expertise. However, it remains unknown whether specific visual expertise confers general changes in perception. Given that 60 to 80% of diagnostic errors are perceptual in nature^6^, the visual perceptual abilities of radiologists and radiographers should be examined. This study uses a visual illusion (VI) task to demonstrate that medical image interpretation abilities may extend beyond that domain of expertise.

Expertise in radiology encompasses deep knowledge of medical imaging, anatomy, and pathology. It relies on radiologists’ advanced visual search patterns and ability to discern critical details in medical images^5^. Here, we refer to radiographers and radiologists as experts, compared to the general public who will be referred to as non-experts. Interpretation of medical images involves a combination of cognitive (analysis and interpretation) and perceptual (visual search, visuospatial abilities) skills^6–8^. Experts outperform non-experts in detecting abnormalities [e.g.,^9–11^], particularly with brief exposure times, ranging from 250 to 2000 milliseconds^12^. With increasing experience, experts in medical image interpretation learn to focus on target-relevant areas while ignoring irrelevant content^13^, resulting in quicker fixations on task-relevant areas^14^. Experts also develop specific expectations about what to look for in an image, suggesting that input from memory enhances their ability to detect abnormalities more rapidly^4^. These findings suggest that, through extensive exposure to specific stimuli, experts in medical image interpretation develop finely tuned visual search skills in their domain of expertise.

Previous theoretical models have proposed that superior perception abilities do not generalise beyond a specific domain of visual expertise [e.g.,^4,15^]. This assertion is underpinned by the belief that experts’ superior performance within their respective domains is afforded by top-down influences. Top-down perception involves perceiving the global picture (seeing the forest before the trees), shaped by prior knowledge and expectations^16^. Experts utilise peripheral and parafoveal vision to analyse extensive portions of an image simultaneously^17–20^, implicating a top-down approach to visual processing. However, previous studies have reported that superior visual abilities conferred by expertise may not generalise beyond specific stimuli. For example, experts are no faster than non-experts at spotting the character Wally (Waldo in the U.S.) or the word NINA among distractors^21^ and, even in tasks superficially resembling medical image searches, experts did not outperform non-experts^22^. Similar findings have been documented in research concerning experts’ visual search abilities (for overview, see^4^) and memory tasks involving visual stimuli, such as objects or scenes^23^.

Research has yet to investigate whether enhancements in experts’ visual perception abilities are a product of specialist professional training^7,24^. Only two studies have addressed this issue. Bass and Chiles^25^ found a general absence of predictive relationships between experts’ domain-general visual abilities (contrast sensitivity or visual acuity) and their ability to spot abnormalities in medical images. Sowden and colleagues^26^ found that, although experts exhibited improved contrast sensitivity, non-experts with no previous experience interpreting medical images also enhanced their ability to discern shade differences after practicing for 10 days. These findings provide mixed evidence about whether radiology training leads to lasting alterations in general visual perception.

In addition to visual search and memory abilities, at least two other skills are required for medical image interpretation: visual context integration and perceptual rescaling. Context integration refers to the ability to visually integrate different elements of a visual scene. With increasing experience, medical image interpretation experts may learn to focus on relevant areas and ignore irrelevant content^13^. Furthermore, successful interpretation of medical images necessitates perceptually transforming a 2D image into a 3D scene, thereby achieving a more lifelike representation of the corresponding part of the human body [e.g.,^27^].

Both the ability to visually disregard illusion-inducing details and perceptual rescaling have been linked to VI susceptibility^28–30^. For example, when no surroundings are presented in the Ebbinghaus illusion, humans can correctly detect size differences of 2% between circles^31^. However, performance drops when a misleading context is applied, potentially due to illusory size differences. This makes VIs a valuable tool in the study of context integration ability. Relatedly, perceptual rescaling plays a pivotal role in accurately estimating the sizes of objects at varying distances in the 3D world^32^. The human visual system automatically rescales identically-sized objects placed at different distances, causing us to perceive them as equally sized, even though they project different visual angles onto the retina. However, these mechanisms can operate inappropriately in images. All the visual stimuli in a 2D image are roughly at the same real depth - the distance between the image and the eye - and perceptual rescaling mechanisms can result in illusory distortions in size perception. These effects are thought to operate in a number of visual illusions, such as the Ponzo, Ebbinghaus, Müller-Lyer, and Shepard’s Tabletops illusions^30,33^ respectively.

Context integration and perceptual rescaling mechanisms may result in illusory size differences, with both processes potentially interfering with judgements of objects in medical images. Acquired expertise through professional training may involve the ability to ignore irrelevant visual context when judging object size, a skill that might be absent amongst nonexperts. If this ability extends beyond the specific domain of medical imaging, we would predict experts in medical image interpretation to show superior size judgement in geometrical visual illusions that derive from inappropriate context integration and perceptual rescaling.

For the first time, we tested whether specific visual expertise induced by professional training affords domain-general perceptual advantages in terms of reduced susceptibility to visual illusions. Experts in medical image interpretation (reporting radiographers, trainee radiologists, and certified radiologists) and a control group consisting of psychology and medical students were presented with the Ebbinghaus, Ponzo, Müller-Lyer, and Shepard Tabletops visual illusions via forced-choice tasks. Participants were tested on their size discrimination, an ability that draws on both context integration and perceptual rescaling [see^30^ and^34^ for reviews]. We hypothesised that experts in medical image interpretation would be less susceptible to VIs, responding more accurately as a result of increasingly localised and stimulus-driven perception conditioned through their acquisition of visual expertise. Crucially, our results will provide insight into whether specific visual expertise elicits by-products for visual perception more broadly, informing existing and future theoretical models of expertise development [e.g.,^4,15^].

## Method

### Participants

Our ‘high visual expertise’ group consisted of trainee radiologists, reporting radiographers, and certified radiologists (*n* = 44; female = 22; non-disclosed = 1; *M* age = 36.01 years, *SD* = 9.45, *M* years of professional experience viewing medical images = 12.12 years, *SD* = 9.20, *M* medical images per day = 78.88, *SD* = 175.77). Of these participants, 10 were recruited from the Norwich Radiology Academy, six were recruited from Cumbria University, and 28 were recruited during the European Congress of Radiology. Our control group consisted of psychology undergraduates, radiography students, and medical students (*n* = 107; Mage = 22.51 years, SD = 7.86; female = 70). Of these participants, 35 were recruited from the University of East Anglia, 50 were recruited from Lancaster University, 12 radiography students from Cumbria University. and 10 medical and radiography students from the European Congress of Radiology. An additional 46 participants who performed below chance level (scores < 3 out of 4) on the control trials for a given illusion (which were designed to detect potential strategy use and lapses in attention) were excluded: 18 psychology undergraduates,

13 medical students, and 15 radiologists and radiographers.

We consider both radiologists and reporting radiographers to be experts in medical image interpretation. Radiologists are practitioners with a medical degree who perform medical image interpretations; reporting radiographers interpret and provide clinical reports on medical images in a similar fashion. Research shows that both radiologists and reporting radiographers have comparable rates for diagnostic accuracy, indicating equivalent levels of visual expertise in the domain of medical image interpretation [e.g.,^35,36^]. Compared to radiography students, radiology trainees are all qualified medical doctors choosing to specialise in the field of radiology and performing more medical image interpretations, hence these were included in the expert group. All participants were naive to the study’s hypotheses and provided informed consent to partake in this study. All procedures performed in this study were in accordance with the ethical standards of institutional and national research committees – the ethical approval was granted by Lancaster University.

### Apparatus and materials

Experts and non-experts were tested on HP Elitebook, HP Omen, and Lenovo ThinkPad laptops with a screen width of 14 inches. The sizing of the illusions on-screen was standardised (i.e., the stimuli were exactly the same dimensions on all laptops and brightness levels were set to maximum on all laptops). The experiment was developed using the computer software EPrime 2.0^37^. Our paradigm was a shortened version of the task developed by Phillips et al.^29^. This task is frequently used to study VI susceptibility across various cultures and populations, including children [e.g.,^31^] and clinical groups [e.g.,^38^].

The experiment consisted of four geometrical VIs: the Ebbinghaus, Ponzo, MüllerLyer, and Shepard Tabletops illusions (see Table 1 for stimuli examples). The Ponzo, Shepard Tabletops, and Müller-Lyer illusions were developed by Chouinard et al.^39^, while the Ebbinghaus illusion was developed by our research team. Examples of all illusions are presented in Table 1. For the Ebbinghaus and Ponzo illusions, the target components of the stimuli (i.e., the manipulated parts of the visual illusion) were coloured orange, while the context was purple. The parts of the stimuli that were not manipulated in the geometrical VIs were held at a constant size of 100 pixels. In the Ebbinghaus illusion, the large and small surroundings had diameters of 150 and 50 pixels, respectively. For the Ponzo illusion, two of the converging lines were 420 pixels long and formed a 64-degree angle (outer lines), while the other two were 380 pixels long and had a 10-degree angle (inner lines). The arrowheads in the Müller-Lyer illusion were set at a 45-degree angle. The rhombuses constituting the Shepard Tabletops illusion were 200 pixels long and 100 pixels wide.

**Table 1:** Visual illusions used in the study.

Illusion	Effect	Picture
Ebbinghaus	The central circle surrounded by smaller outer circles is usually perceived as bigger.	
Ponzo	The top line is perceived to be longer despite being the same length as the bottom line.	
Müller-Lyer	The line with the arrowheads pointing outwards is seen as longer than the line with arrowheads pointing inwards.	
Shepard Tabletops	The vertical parallelogram is perceived as longer and broader, despite them both being identical in size.	

## Procedure

Participants were seated in front of a computer and instructed to keep an upright posture to maintain the same viewing perspective across the whole experiment. The participant’s face was roughly 60 cm from the screen, ensured by asking each participant to sit so their stomach was always touching the edge of the desk. Participants were informed that they would be presented with a battery of VIs. They were instructed not to try to ‘see through the illusions’ and respond based on their first impression as quickly and accurately as they could. Finally, participants were told that if they were unsure about their answer, they should guess. Most participants were seated in a cubicle. Participants recruited during the European Congress of Radiology sat at a table in a corridor, where other members of the congress could freely pass.

Before completing the experiment, participants provided basic demographic data. Each VI had its own unique set of instructions presented on screen. For the Ebbinghaus illusion, participants had to select the larger circle by pressing the corresponding key on the keyboard.

For the Ponzo illusion, participants had to select the longer of the two horizontal lines. For the Müller-Lyer illusion, the participant had to choose the longer of the two lines (with the instruction that they should focus on the lines between the arrowheads only). For the Shepard Tabletops illusion, the participant had to choose the wider of the two tables. All illusions except the Ponzo illusion were counterbalanced by reversing the images, so the targets appeared on both sides of the screen. All trials for a given illusion were delivered in a block consecutively in a random order. There were 24 trials per illusion divided into six varying difficulty levels.

One difficulty level served as a control, where the context was designed to be helpful (congruent with the illusory effect). For example, in the context of the Ebbinghaus and the Shepard Tabletop illusions, the ‘perceived as larger’ circle/rhombus was actually 2% larger than the comparison circle/rhombus. For the Ponzo and Müller-Lyer illusions, this difference was 4%. The other five trial types were designed to be misleading, where the difference between the two targets varied by 2%, 6%, 10%, 14%, and 18% for the Ebbinghaus and Shepard Tabletops illusions, or 4%, 12%, 20%, 28%, and 36% for the Ponzo and Müller-Lyer illusions. On each trial, correctly identifying the longer/larger stimuli was scored 1 while identifying the incorrect stimuli was scored 0. Thus, participants could score a maximum of 20 correct answers per illusion (excluding control trials), with higher scores indicating lower susceptibility to VIs.

### Analytic plan and design

Firstly, the normality of the data set was assessed, and outliers were handled using the winsorising technique^40^; rather than omitting outliers altogether, they were replaced with the closest non-outlier value from the sample^41^. The method is known for its robustness and simplicity^40^. The data for each participant were analysed excluding scores from the control condition, which was used to detect lapses in attention and/or strategy use. Response accuracy data were analysed using generalised linear mixed-effects models using the glmer function from the lme4 package in R^42^. Response was the dependent variable. Lastly, differences in illusion susceptibility scores were assessed using JASP^43^ via a 2 (group: radiography students vs. psychology students) x 4 (Illusion type: Ebbinghaus, Ponzo, Müller-Lyer, and Shepard Tabletops) repeated measures ANOVA to investigate if there are any pre-existing, superior visual abilities in individuals pursuing careers in medical image interpretation.

## Results

### Outliers

In the control group, one overall value for the Müller-Lyer illusion was decreased via winsorising from 18 to 15, and two were increased from eight to 10. Following these adjustments, the normality of the data was assessed using the Shapiro-Wilk test across each VI within each of the two groups. These tests indicated normal distribution for three VIs in the expert group (*p* > .104), except for the Müller-Lyer illusion (*p* = .007). For the control group, VIs were not normally distributed (*p* < .019), except the Ponzo illusion (*p* = .124).

### Generalised linear mixed effect models

Stimuli size differences (%) between comparison stimuli were coded as 2, 6, 10, 14, and 18 for the Ebbinghaus and Shepard Tabletops illusions, and 4, 12, 20, 28, and 36 for the Müller-Lyer and Ponzo illusions. The dependent variable was response accuracy – for each trial per VI the participant could score 0 (incorrect answer) or 1 (correct answer). The likelihood of responding correctly by chance was 50%. The baseline model contained a by-participant random intercept with a random slope of stimuli size difference. Fixed effects of expertise group and stimuli size difference were tested individually, then in combination, and then the interaction between these effects was tested. Each increasingly complex model was compared against the baseline or current best-fitting model to test whether the additional effects significantly improved fit. Once the final model containing experimental variables was established, individual difference measures were added to test whether their inclusion significantly improved fit (age as a numerical value, and sex scored categorically – males were coded as -0.5, while females were coded as 0.5) in the combined population, then years of experience and images per day in the expert group only (also as numerical values). Only the final models are reported below (see Table 2); model-building sequences for each illusion are detailed in the Supplementary Materials.

#### The Ebbinghaus illusion

**Fig. 1:**
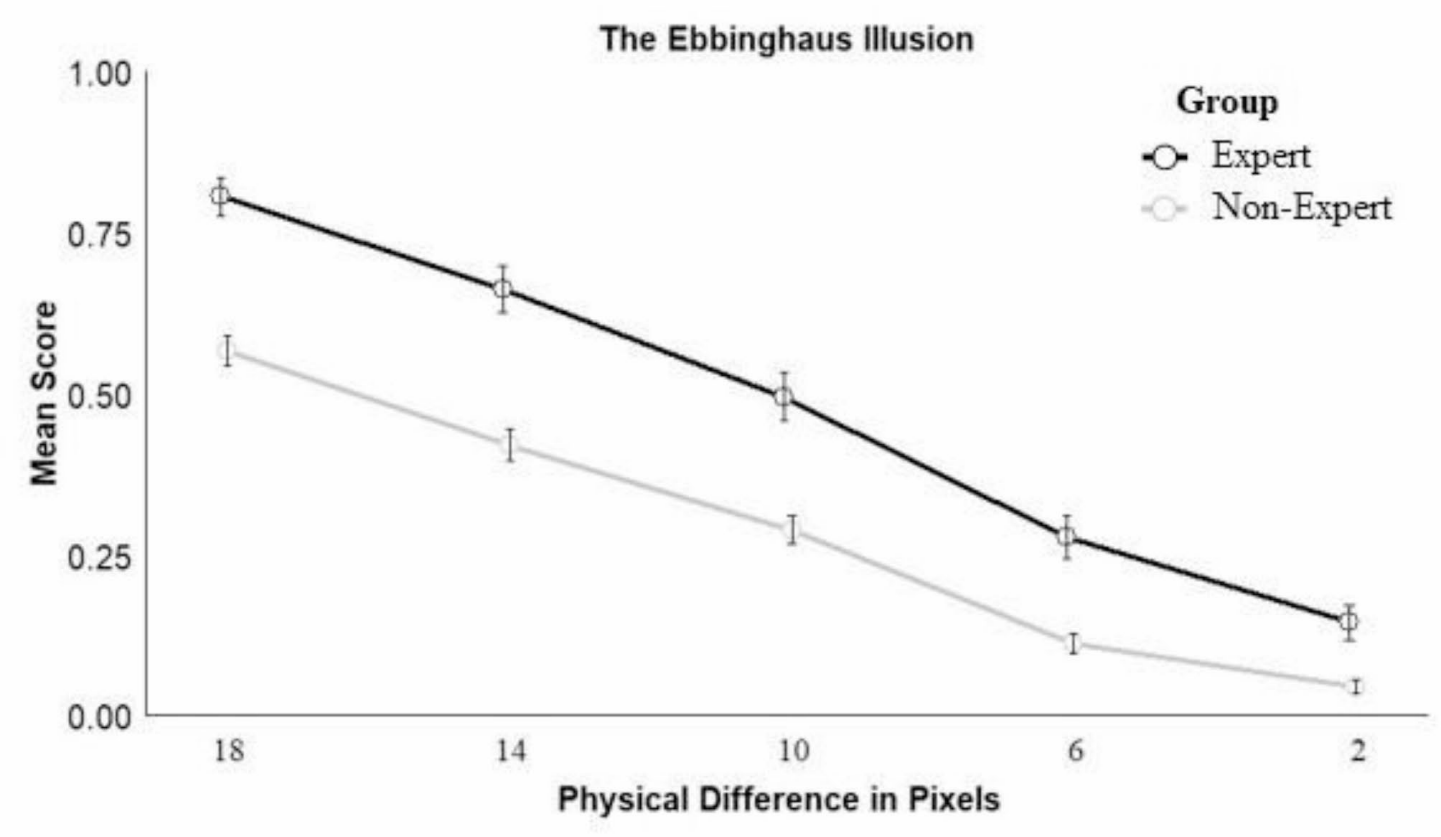
Participants’ responses across different conditions for the Ebbinghaus Illusion. *Note*: The larger the physical difference between the target and the comparison circle, the weaker the illusion.

The best-fitting model for the Ebbinghaus illusion included fixed effects of size difference (*z* = -19.85, *p* < .001) and group (*z* = 4.25, *p* < .001). Across groups, participants were more likely to respond accurately as differences between stimuli increased. Experts (*M* = 0.49, *SD* = 0.43) were more likely to respond accurately across difficulty levels than nonexperts (*M* = 0.29, *SD* = 0.36; see Fig. 1).

When exploring the effects of individual differences, the inclusion of sex significantly improved model fit. Across groups, males responded significantly more accurately than females (*z* = -2.09, *p* = .037). This replicates previous research on sex differences in susceptibility to the Ebbinghaus illusion (e.g., Phillips et al., 2004). Age was not a significant predictor. For the group with expertise in medical image interpretation, the inclusion of medical images viewed per day or years of experience as fixed effects did not significantly improve model fit.

#### The Ponzo illusion

**Fig. 2:**
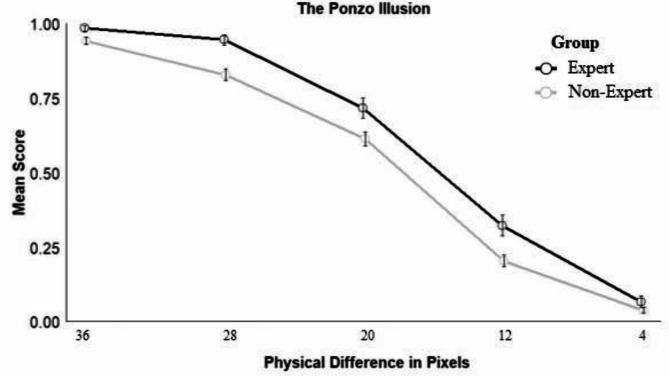
Responses across different conditions for the Ponzo Illusionn. *Note*: The larger the physical difference between the target and the comparison line, the weaker the illusion.

The best-fitting model for the Ponzo illusion included fixed effects of size difference (*z* = 24.60, *p* < .001) and group (*z* = 2.54, *p* = .011). Across groups, participants were more likely to respond accurately as differences between stimuli increased. Experts (*M* = 0.61, *SD* = 0.46) were more likely to respond accurately across difficulty levels than non-experts (*M* = 0.52, *SD* = 0.47; see Fig. 2).

When exploring individual differences, the inclusion of sex significantly improved model fit. Across groups, males responded significantly more accurately than females (*z* = 2.28, *p* = .017). This replicates previous research on sex differences in susceptibility to the Ponzo illusion (e.g., Miller, 2001). Age was not a significant predictor. For the group with expertise in medical image interpretation, the inclusion of medical images viewed per day or years of experience as fixed effects did not significantly improve model fit.

#### The Müller-lyer illusion

**Fig. 3:**
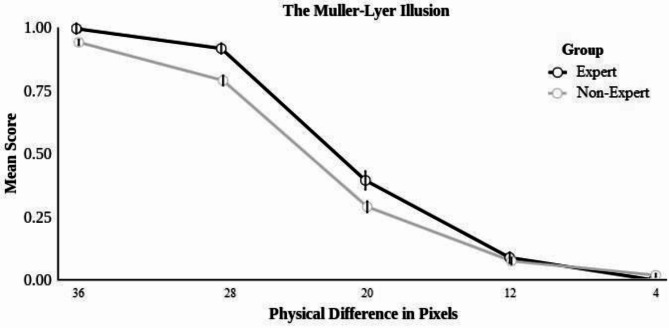
The interaction across different conditions and group for the müller-lyer illusion. *Note*: The larger the physical difference between the target and the comparison line, the weaker the illusion.

For the Müller-Lyer illusion, the best-fitting model included the group x size difference interaction (*z* = -2.90, *p* = .002; see Fig. 3). The interaction was deconstructed by testing the effect of size difference for experts and non-experts separately. The effect of size difference was significant for both the expert (*z* = -15.70. *p* < .001) and non-expert groups (*z* = -23.13, *p* < .001); both groups were more likely to respond correctly as size differences between stimuli increased. We also tested the effect of the group for trials with low (4–20%) and high differences (28–36%) in stimuli size separately. While the groups’ response accuracy did not significantly differ when size differences between stimuli were small (*z* = 0.02, *p* = .988), experts (*M* = 0.96, *SD* = 0.21) responded with significantly greater accuracy than non-experts.

(*M* = 0.87, *SD* = 0.34) when size differences between stimuli were larger (*z* = 3.60, *p* < .001). This suggests that experts were more accurate in their ability to discern the length of the two lines as that difference becomes more evident compared to non-experts. When exploring the effects of individual differences, the inclusion of either sex, age, images viewed per day, or age of expertise did not significantly improve model fit.

#### The Shepard Tabletops illusion

**Fig. 4:**
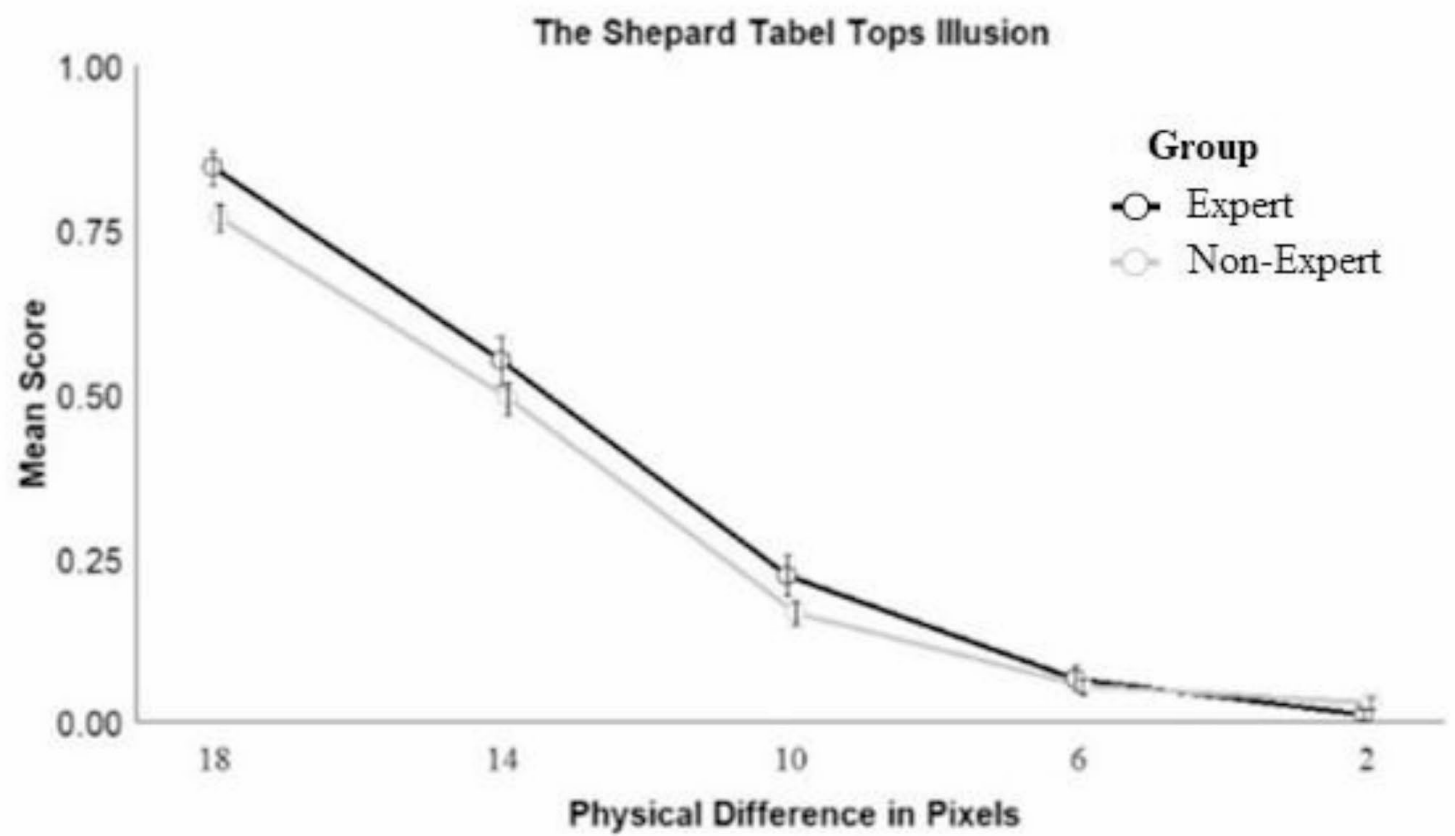
Responses across different conditions for the Shepard Tabel Tops Illusion. *Note*: The larger the physical difference between the target and the comparison rhombus, the weaker the illusion.

The final model included the fixed effect of size difference (*z* = -21.20, *p <* .001; see Fig. 4), indicating that participants were more likely to respond accurately as differences between stimuli increased. When exploring the effects of individual differences, the inclusion of sex, age, or age of expertise did not significantly improve model fit. Including medical images viewed per day improved model fit, however, the effect was not significant (*z* = -1.44, *p* = .149)..

**Table 2:** Generalised Linear models for all four visual illusions.

Visual illusion	Fixed effects	Estimated coefficient	Standard error	z	Pr(>|z|)
Ebbinghaus	(Intercept)	-4.0	0.2	-17.8	< 0.001
	Group	1.5	0.4	4.3	< 0.001
	Difference	-0.3	< 0.1	-19.9	< 0.001
		AIC	BIC	logLik	Deviance
		2481.5	2517.6	-1234.7	2469.5
Ponzo	(Intercept)	-5.1	0.2	-20.7	< 0.001
	Group	0.8	0.3	2.5	= 0.011
	Difference	-0.3	0.1	-24.6	< 0.001
		AIC	BIC	logLik	Deviance
		2011.9	2048.0	-1000.0	1999.9
Müller-Lyer	(Intercept)	-7.3	0.4	-20.1	< 0.001
	Group	-1.3	0.7	-1.9	= 0.069
	Difference	-0.3	< 0.1	-20.9	< 0.001
	Group x Difference	-0.1	< 0.1	-2.9	= 0.004
		AIC	BIC	logLik	Deviance
		1863.1	1905.1	-924.5	189.0
Shepard Tabletops	(Intercept)	-6.3	0.3	-22.8	< 0.001
	Difference	-0.5	< 0.1	-21.2	< 0.001
		AIC	BIC	logLik	Deviance
		2209.5	2239.6	-1099.8	2199.5

### Radiography students versus psychology students

Finally, to show that observed group differences in VI susceptibility are unlikely to be caused by pre-existing superior visual abilities, we compared radiography students (*n* = 12; *M*age = 31.42, *SD* = 9.56; female = 10) and psychology students (*n* = 12, *M* age = 25.53, *SD* = 11.02, female = 10), who did not differ on age (*p* = .180). There was a main effect of illusion, *F*(3, 66) = 6.27, *p* < .001, ηp2 = 0.22, but no effect of group, *F*(1, 22) = 1.04, *p* = .318, ηp2 = 0.05, and no interaction, *F*(3, 66) = 0.05, *p* = .985, ηp2 < 0.01. This suggests that our expert sample’s reduced susceptibility to VIs in the preceding analyses may be attributable to extensive training and experience, rather than superior perceptual abilities prior to acquiring visual expertise.

## Discussion

To discover whether specific visual expertise affords general benefits to visual perception, we investigated whether radiologists and reporting radiographers—professionals with extensive exposure to medical imagery—are less susceptible to basic visual illusions than individuals who lack similar training. Consistent with our hypothesis, experts in medical image interpretation demonstrated reduced susceptibility to the Ebbinghaus, Ponzo, and Müller-Lyer illusions, but not the Shepard Tabletops illusion. As medical radiography students performed similarly to psychology students, it seems unlikely that these differences in VI susceptibility are due to pre-existing visual abilities. These findings present evidence that expertise in perceiving specific kinds of visual stimuli may afford domain-general benefits to visual perception. Our results diverge from previous literature and existing models of perceptual expertise [e.g.,^4^], which have claimed that proficiency does not transfer beyond the specific domain of expertise.

Our findings challenge existing claims about the domain-specific, or even sub-domain specific, nature of experts’ visual abilities in the field of medical image interpretation (e.g.,^4,19^]. The holistic processing account^4^ posits that an individual’s enhanced ability to interpret medical images should not translate into improved performance in other areas. Indeed, experts typically do not outperform laypersons in visual search tasks beyond their area of expertise (for an overview, see^4^). However, in contrast to most research within the field of visual expertise, our study did not assess visual search abilities. Instead, we evaluated experts’ ability to detect small changes in size within the context of visual illusions. This approach tapped into different perceptual abilities to those involved in visual search tasks, and our findings suggest that expertise in medical image interpretation may improve the ability to disregard irrelevant context and enhance perceptual rescaling abilities. The ability to disregard irrelevant context is crucial for the successful interpretation of medical images^13^, while perceptual rescaling (which causes VIs susceptibility in some illusions) may be required to turn 2D images into 3D representations of the human body, which experts in radiology frequently do^7^. Therefore, extensive practice in attending to task relevant areas, combined with turning 2D into 3D, may result in perceptual changes that are transferrable beyond the domain of expertise. Overall, these data are the first to demonstrate that professional visual expertise may induce changes in visual perception that extend beyond a specific domain.

To explain these results, we propose that a stronger local bias is a by-product of extensive visual expertise. Previous studies have shown that radiology experts are quick to fixate on task-relevant areas in medical images, thereby improving the speed at which they detect abnormalities^4^. This efficiency has been linked to memorised representations of target areas^44^, implicating stronger top-down influences, where previously seen medical images and expectations about the appearance of healthy scans direct attention to relevant areas where abnormalities can be found. However, we argue that this approach could also include a local component – experts may demonstrate the ability to visually disregard irrelevant areas of the image by focusing on local details of the visual scene^13^.

When instructed to discriminate the sizes of two stimuli, our expert sample may have processed context to a lesser extent, thereby reducing the illusory effect and resulting in more accurate estimates. This effect appeared to be particularly evident in the Ebbinghaus illusion, where the illusion-inducing elements are not physically incorporated into the targets (i.e., the inner circles), allowing the context to be visually ignored. The possibility that the observed effect of visual expertise is due to reduction of top-down influences is supported by the fact that VI susceptibility does not rely on previous knowledge (e.g. being told how the VI works, does not remove its effect). Prior research has also demonstrated that experts’ eye movement patterns are significantly influenced by local stimulus effects relative to non-experts^45^, indicating superior ability to focus on areas of interest. Thus, our findings suggest that the role of local biases should be acknowledged and integrated into current theories of perceptual expertise.

One possibility is that top-down influences *and* a local processing bias develop simultaneously through training the visual system on specific stimuli. As knowledge and target representations (top-down) develop during the acquisition of expertise, the ability to focus on local areas of the image while suppressing irrelevant information also develops, enhancing target detection^13,14^. This theory also explains why experts in radiology do not retain their superior visual search abilities outside their area of expertise (like searching for Waldo/Wally character^21^). Visual search is primarily driven by top-down influences, which do not translate to finding targets beyond one’s area of expertise, as mental target representations cannot be applied. This theory requires validation in other domains of visual expertise, such as chess.

Research indicates there is no unique common mechanism underpinning VIs^46^. With experts showing the strongest reduction in their susceptibility to the Ebbinghaus as compared to the control group (as suggested by the largest estimated coefficient of 1.5 compared to other VIs), it would be logical to look at radiological training for a possible explanation. The Ebbinghaus is an illusion of relative size perception, and an expert is routinely required to comment on the size of image features when writing radiological reports of image findings. These are often objective quantitative measurements of features such as blood vessel diameter, the diameter of a tumour, or size of an acute stroke on a brain scan for example. It is important that these measurements are accurate for diagnostic purposes so all imaging software will include calibrated calipers to ensure accuracy. It is probable that perceptual learning will be happening, as the expert on initial viewing of the image may detect that the size of an organ or blood vessel could be outside the normal range, but they will get instant feedback when they use the software to obtain an accurate measurement. This suggests that advantages in ignoring irrelevant context may develop through profession-specific training.

We observed significant differences between experts and non-experts on three out of four VIs (the Ebbinghaus, Ponzo, and Müller-Lyer illusions), but not the Shepard Tabletops illusion. Unlike the other VIs, the Shepard Tabletops illusion does not present misleading context – its illusory effect comes from differences in orientation of the two rhombuses. Therefore, in line with our hypothesis that experts would show superior ability to ignore irrelevant context, the lack of significant differences between the two groups is unsurprising. With no misleading context to ignore, experts did not benefit from heightened attention to task relevant areas. Alternatively, the Shepard Tabletops illusion produced the lowest susceptibility scores of all the illusions tested, perhaps indicating a generally increased difficulty in responding to this illusion. If the difficulty level was decreased (e.g., by increasing the intervals by which the conditions were varied), experts could exhibit reduced susceptibility, in line with the other illusions. This, however, requires further investigation.

Importantly, VI susceptibility did not significantly differ between radiography and psychology students. This is indirect evidence that individuals who pursue careers in medical image interpretation do not self-select based on inherent visual abilities; instead, these abilities most plausibly develop through practice. Further research is required to elucidate what components of radiology training are responsible for reducing susceptibility to VIs and how much exposure to training is required to elicit changes in perception. Lastly, as reported in previous studies, we observed that susceptibility to the Ebbinghaus and Ponzo illusions is diminished in males [e.g.,^29,47^]. This finding suggests that, under specific illusory conditions, context integration may be reduced in males due to possible differences in local processing. Future studies investigate the role of sex differences upon VI susceptibility, as clear evidence on this issue is lacking^48^.

Future studies should include other groups considered experts in visual perception, such as chess players, as well as conduct comparisons between different sub-domains of radiology expertise. Such investigations would elucidate whether different sub-domains of expertise (e.g., chest imaging versus mammography) and their associated training differentially affect visual perception. It is of particular interest, as Nodine and Mello-Thoms^19^ note that gaining expertise in interpreting chest images does not automatically apply to one’s ability to interpret mammograms – suggesting that the ability to interpret medical images is even subdomain-specific.

## Conclusion

Our research advances theoretical understanding of how expertise and training impact fundamental mechanisms underpinning visual perception. Current models of visual expertise claim that enhanced top-down influences result from visual training and developing expertise. Focusing on perceptual skills that do not correspond to visual search capabilities, we present evidence that experts may learn to attend to visual scenes locally, thus disregarding irrelevant context. For this reason, some visual skills developed by experts in radiology and radiography appear to be transferrable beyond their domain of expertise.

## Electronic supplementary material

Below is the link to the electronic supplementary material.

Supplementary Material 1

## Data Availability

The data described in this paper can be obtained upon request from the first author at r.wincza@gmail.com.
