# Correction: Specific visual expertise reduces susceptibility to visual illusions

**DOI:** 10.1038/s41598-025-06746-8

**Published:** 2025-06-17

**Authors:** Radoslaw Wincza, Calum Hartley, Tim Donovan, Sally Linkenauger, Trevor Crawford, Debra Griffiths, Martin Doherty

**Affiliations:** 1 Lancaster University, Lancaster, UK; 2 University of Central Lancashire, Preston, UK; 3 Cumbria University, Lancaster, UK; 4 University of East Anglia, Norwich, UK

Correction to: *Scientific Reports* 10.1038/s41598-025-88178-y, published online 13 March 2025

The original version of this Article contained an error in Table 1, where the effects for the ‘Muller-Lyer’ and the ‘Shepard Tabletops’ illusions were interchanged.

The original Article has been corrected.

