# Supplementary material for: Specific visual expertise reduces susceptibility to visual illusions
Source: Sci Rep. 2025 Mar 13;15:5948. doi: 10.1038/s41598-025-88178-y (PMC11904176; doi:10.1038/s41598-025-88178-y)
Supplement: Supplementary file 1 — Supplementary Material 1 [file 41598_2025_88178_MOESM1_ESM.pdf]

Supplementary Materials – Model Building Sequences

General Information

Non-experts were coded as -0.5 while experts were coded as 0.5. Stimuli size difference (%) between comparison stimuli was coded as 2, 6, 10, 14, and 18 for the Ebbinghaus and Shepard Tabletops illusions, and 4, 12, 20, 28, and 36 for the Muller-Lyer and Ponzo illusions. The dependent variable was response accuracy – for each trial per VI the participant could score 0 (incorrect answer) or 1 (correct answer). The likelihood of responding correctly was 50%. Data were analysed via generalised linear mixed effects models in R (R Core Team, 2019) using the glmer function from the lme4 package (Bates et al., 2015).

Ebbinghaus Illusion

The baseline model (Model 1) contained a by-participant random intercept with a random slope of stimuli size difference (z = -18.36, p < .001). Then, the fixed effect of the group was added (Model 2), significantly improving the fit compared to the baseline model (Model 1: χ^2 = 11.92, p < .001). We then added the stimuli size difference (Model 3) which improved the model fit over the baseline model (Model 1: χ^2 = 183.16, p < .001). Furthermore, we included both effects combined (Model 4), which also improved the model fit over the previous two models (Models 2 and 3; χ^2 = 188.35, p < .001; χ^2 = 17.11, p < .001, respectively). Finally, adding an interaction effect between stimuli size difference and group (Model 5) did not significantly improve the model (Model 4: χ^2 = 1.01, p < .315).

While testing individual differences, adding age (Model 6) did not significantly improve the best-fitting model (Model 3; χ^2 = 1.72, p = .190), while adding sex as a factor (Model 7) marginally improved it (Model 7: χ^2 = 4.27, p = .039). Both years of experience (Model 8) and images per day (Model 9) failed to improve the base-fitting model (Model 3; χ^2 < 0.01, p = .948; χ^2 < 0.01, p = .978).

Ponzo Illusion

The baseline model (Model 1) contained a by-participant random intercept with a random slope of stimuli size difference (z = -11.13, p < .001). Then, the fixed effect of the group was added (Model 2), significantly improving the fit compared to the baseline model (Model 1: χ^2 = 4.69, p = .030). We then added the stimuli size difference (Model 3) which improved the model fit over the baseline model (Model 1: χ^2 = 282.34, p < .001). Furthermore, we included both effects combined (Model 4), which also improved the model fit over the previous two models (Model 2 and 3: χ^2 = 283.88, p < .001; χ^2 = 6.23, p = .013, respectively). Finally, adding an interaction effect between stimuli size difference and group (Model 5) did not significantly improve the model (Model 4: χ^2 = 2.35, p < .125).

While testing individual differences, adding age (Model 6) did not significantly improve the best-fitting model (Model 4: χ^2 =1.21, p = .272), while adding sex as a factor (Model 7) also improved the model (Model 4: χ^2 = 5.56, p = .018). Both years of experience and images per day (Models 8 and 9) failed to improve the best-fitting model (Model 4: χ^2 = 0.01, p = .815; χ^2 = 1.84, p = .178).

Muller-Lyer Illusion

The baseline model (Model 1) contained a by-participant random intercept with a random slope of stimuli size difference (z = -18.36, p < .001). Then, the fixed effect of the group (Model 2) was added, significantly improving the fit compared to the baseline model (Model 1: χ^2 = 15.44, p < .001). We then added the stimuli size difference (Model 3) which improved the model fit over the baseline model (Model 1: χ^2 = 282.45, p < .001). Furthermore, we included both effects combined (Model 4), which also improved the model fit over the previous two models (Models 2 and 3; χ^2 = 279.36, p < .001; χ^2 = 12.35, p < .001, respectively). Finally, adding an interaction effect between stimuli size difference and group (Model 5)

significantly improved the model (Model 4: $\chi^2 = 9.93$, $p = .002$). The interaction is deconstructed in the main text.

While testing individual differences, adding age (Model 6) did not significantly improve the best-fitting model ($\chi^2 = 0.14$, $p = .713$), while adding sex as a factor (Model 7) also improved the model ($\chi^2 = 0.47$, $p = .495$). Both years of experience and images per day failed to improve the best-fitting model (Models 8 and 9: $\chi^2 = 0.62$, $p = .431$; $\chi^2 = 1.95$, $p = .163$).

Shepard Tabletops Illusion

The baseline model (Model 1) contained a by-participant random intercept with a random slope of stimuli size difference ($z = -18.36$, $p < .001$). Then, the fixed effect of the group was added (Model 2), which did not significantly improve the fit compared to the baseline model (Model 2: $\chi^2 = 1.08$, $p = .300$). We then added the stimuli size difference (Model 3) which improved the model fit over the baseline model (Model 1: $\chi^2 = 234.98$, $p < .001$). Including both factors together (Model 4) improved the model fit over the group-only model (Model 2: $\chi^2 = 235.14$, $p < .001$) but not the stimuli size difference-only model (Model 3: $\chi^2 = 1.24$, $p = .265$). Finally, adding an interaction effect between stimuli size difference and group (Model 5) did not significantly improve the model (Model 3: $\chi^2 = 3.37$, $p < .186$).

While testing individual differences, adding age (Model 6) did not significantly improve the best-fitting model (Model 3: $\chi^2 =1.53$, $p = .465$), while adding sex as a factor (Model 7) also did not improve the model (Model 3: $\chi^2 =2.55$, $p = .279$). Years of experience (Model 8) failed to improve the model (Model: $\chi^2 = 0.61$, $p = .433$) and images per day (Model 9) improved the best-fitting model (Model 3: $\chi^2 = 4.82$, $p = .028$).

Pairwise Comparisons

For experts, the Ebbinghaus illusion's scores ($M = 9.16$, $SD = 5.16$) significantly differed from Ponzo's ($M = 12.05$, $SD = 3.00$; $t(44) = -4.40$, $p < .001$, $d = -0.64$, $BF_{10} = 193.60$)

and Shepard's (M = 6.86, SD = 3.15; $t(44)$ = 3.50, p = .006, d = 0.42, BF_{10} = 4.81), but not the Müller-Lyer's (M = 9.55, SD = 1.78; $t(44)$ = -0.59; p = 1, d = - 0.07, BF_{10} = 0.18). Furthermore, Ponzo's scores (M = 12.05, SD = 3.00) differed significantly from Müller-Lyer's scores (M = 9.55, SD = 1.78; $t(44)$ = 3.81; p = .002, d = 0.66, BF_{10} = 283.45) and Shepard's scores (M = 6.86, SD = 3.15; $t(44)$ = 7.90, $p < .001$, d = 1.43, BF_{10} = $1.914x10^{+9}$). Finally, scores for the Müller-Lyer's scores (M = 9.55, SD = 1.78) differed significantly from Shepard's (M = 6.86, SD = 3.15; $t(44)$ = 4.09, $p < .001$, d = 0.74, BF_{10} = 1541.84). For the non-experts, the Ebbinghaus illusion's scores (M = 5.75, SD = 4.61) significantly differed from Ponzo's (M = 10.51, SD = 3.36; $t(107)$ = -11.38, $p < .001$, d = -0.97, BF_{10} = $1.223x10^{+14}$) and Müller-Lyer's (M = 8.49, SD = 1.76; $t(107)$ = -0.51, $p < .001$, d = -0.55, BF_{10} = 109617.46), but not the Shepard's (M = 5.99, SD = 2.69; $t(44)$ = -0.58; p = 1, d = - 0.05, BF_{10} = 0.12). Furthermore, Ponzo's scores (M = 10.51, SD = 3.36) differed significantly from Müller-Lyer's scores (M = 8.49, SD = 1.76; $t(44)$ = 4.80; $p < .001$, d = 0.53, BF_{10} = 52167.46) and Shepard's scores (M = 5.99, SD = 2.69; $t(44)$ = 10.74, $p < .001$, d = 1.21, BF_{10} = $3.304x10^{+19}$). Finally, scores for the Müller-Lyer's scores (M = 8.49, SD = 1.76) differed significantly from Shepard's (M = 5.99, SD = 2.69; $t(44)$ = 5.94, $p < .001$, d = 0.76, BF_{10} = $2.105x10^{+9}$).
